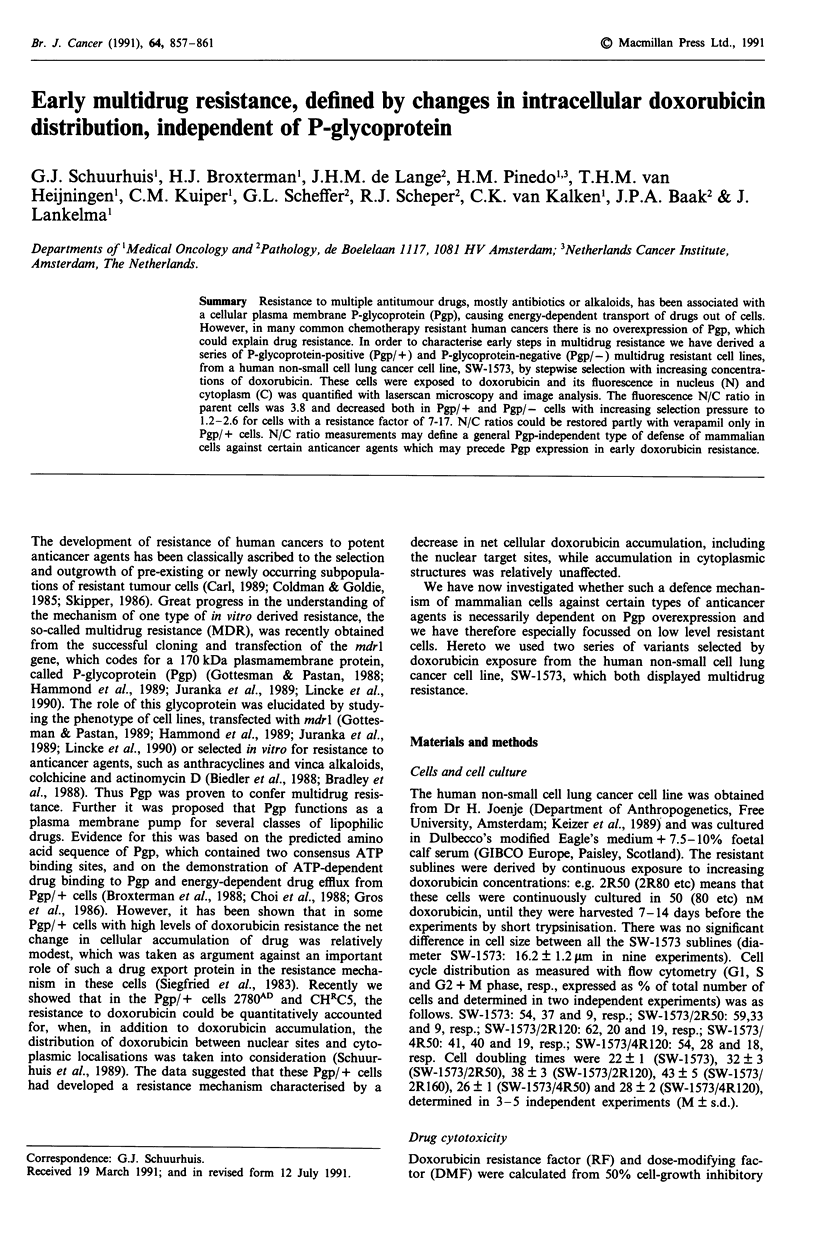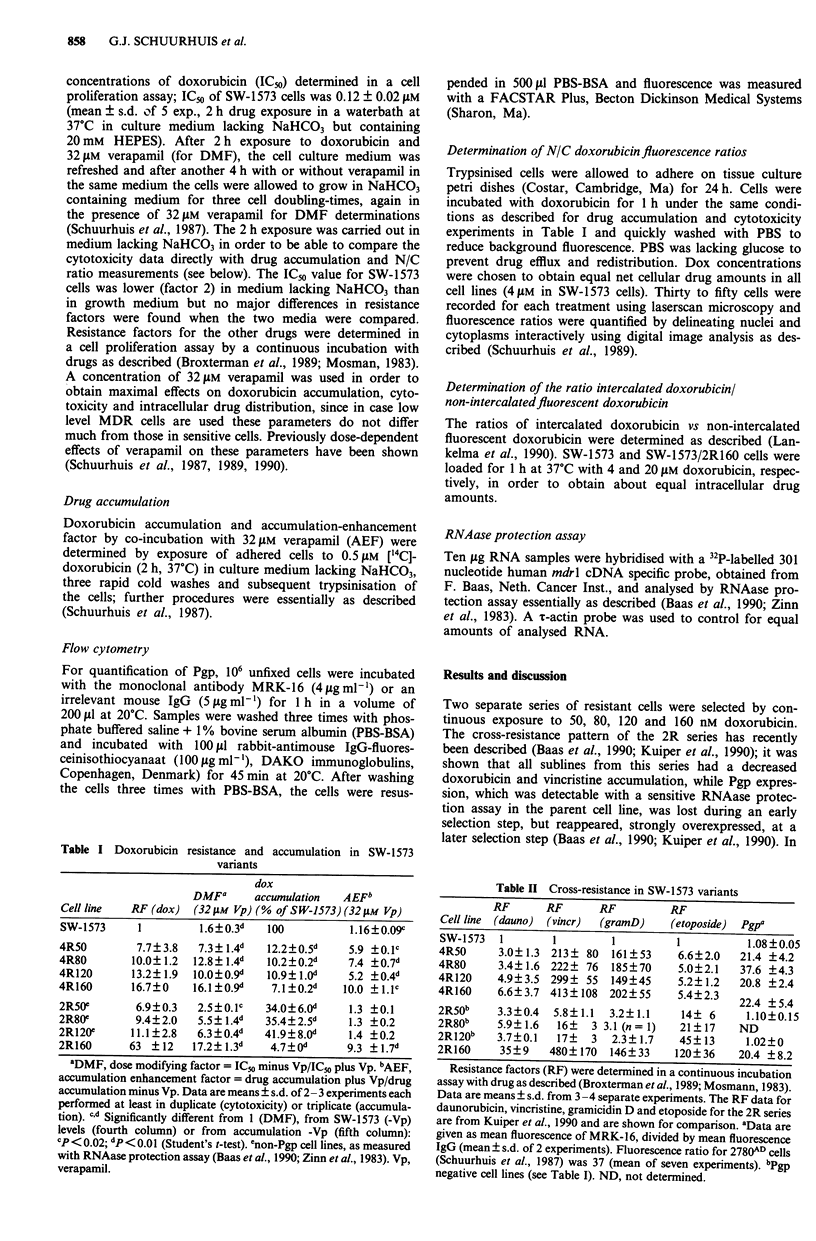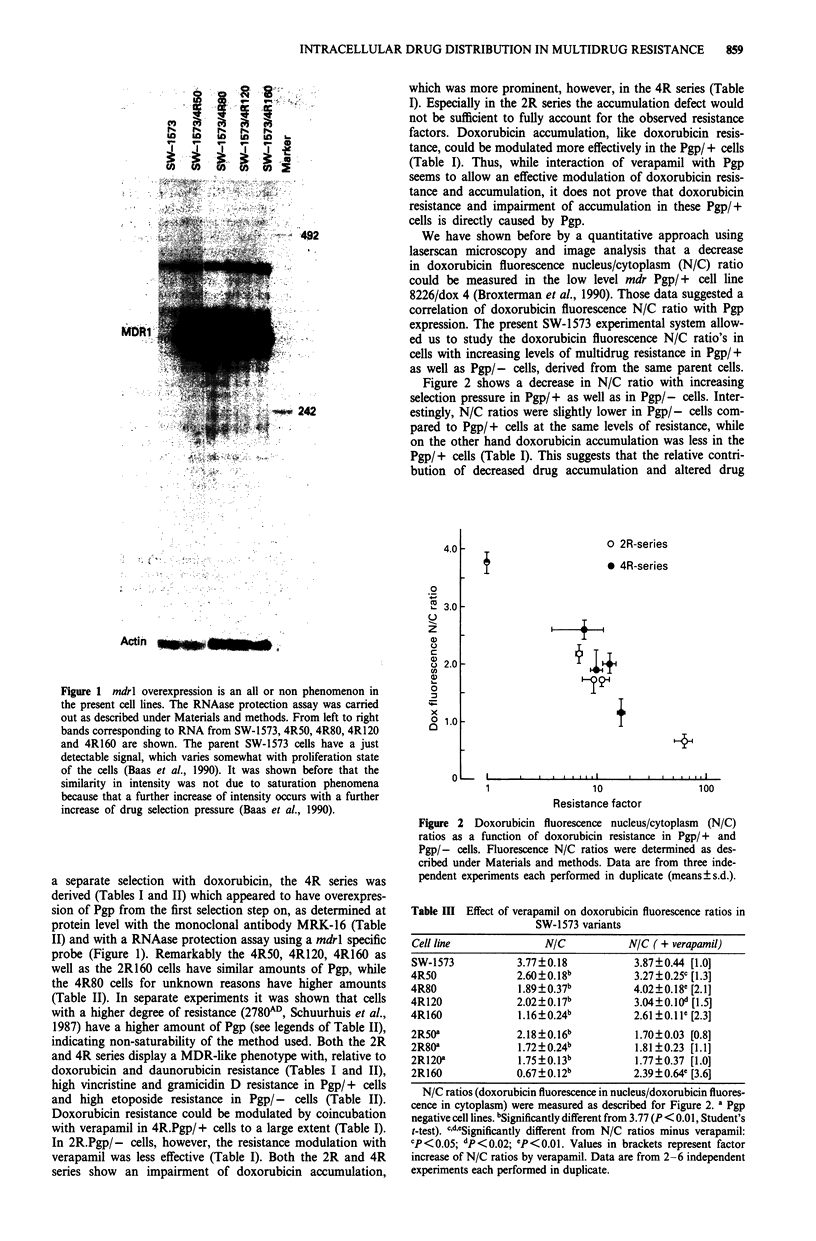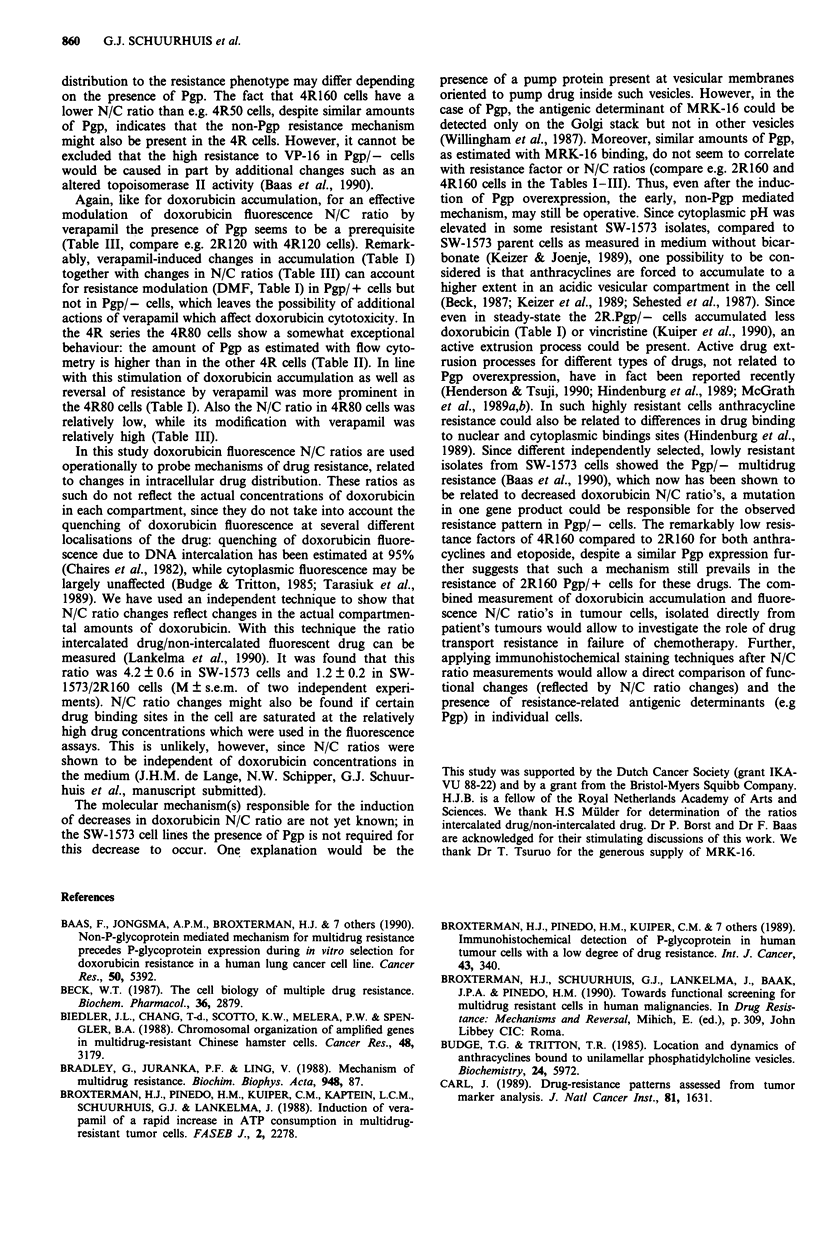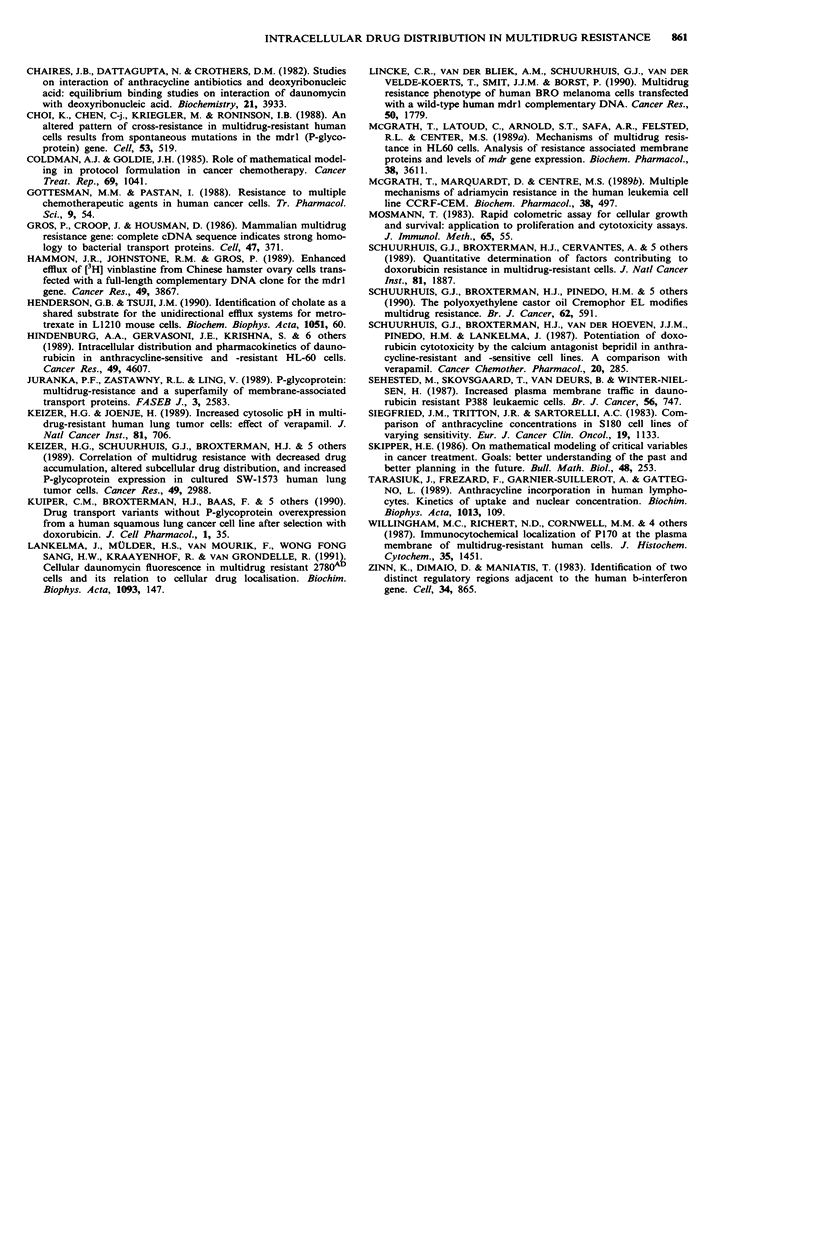# Early multidrug resistance, defined by changes in intracellular doxorubicin distribution, independent of P-glycoprotein.

**DOI:** 10.1038/bjc.1991.413

**Published:** 1991-11

**Authors:** G. J. Schuurhuis, H. J. Broxterman, J. H. de Lange, H. M. Pinedo, T. H. van Heijningen, C. M. Kuiper, G. L. Scheffer, R. J. Scheper, C. K. van Kalken, J. P. Baak

**Affiliations:** Department of Medical Oncology, Amsterdam, The Netherlands.

## Abstract

**Images:**


Br. J. Cancer (1991), 64, 857-861 © Macmillan Press Ltd., 1991

### Early multidrug resistance, defined by changes in intracellular doxorubicin distribution, independent of P-glycoprotein

G.J. Schuurhuis[1], H.J. Broxterman[1], J.H.M. de Lange[2], H.M. Pinedo[1,3], T.H.M. van Heijningen[1], C.M. Kuiper[1], G.L. Scheffer[2], R.J. Scheper[2], C.K. van Kalken[1], J.P.A. Baak[2] & J. Lankelma[1]

*Departments of [1]Medical Oncology and [2]Pathology, de Boelelaan 1117, 1081 HV Amsterdam; [3]Netherlands Cancer Institute, Amsterdam, The Netherlands.*

**Summary** Resistance to multiple antitumour drugs, mostly antibiotics or alkaloids, has been associated with a cellular plasma membrane P-glycoprotein (Pgp), causing energy-dependent transport of drugs out of cells. However, in many common chemotherapy resistant human cancers there is no overexpression of Pgp, which could explain drug resistance. In order to characterise early steps in multidrug resistance we have derived a series of P-glycoprotein-positive (Pgp/+) and P-glycoprotein-negative (Pgp/−) multidrug resistant cell lines, from a human non-small cell lung cancer cell line, SW-1573, by stepwise selection with increasing concentrations of doxorubicin. These cells were exposed to doxorubicin and its fluorescence in nucleus (N) and cytoplasm (C) was quantified with laserscan microscopy and image analysis. The fluorescence N/C ratio in parent cells was 3.8 and decreased both in Pgp/+ and Pgp/− cells with increasing selection pressure to 1.2-2.6 for cells with a resistance factor of 7-17. N/C ratios could be restored partly with verapamil only in Pgp/+ cells. N/C ratio measurements may define a general Pgp-independent type of defense of mammalian cells against certain anticancer agents which may precede Pgp expression in early doxorubicin resistance.

The development of resistance of human cancers to potent anticancer agents has been classically ascribed to the selection and outgrowth of pre-existing or newly occurring subpopulations of resistant tumour cells (Carl, 1989; Coldman & Goldie, 1985; Skipper, 1986). Great progress in the understanding of the mechanism of one type of *in vitro* derived resistance, the so-called multidrug resistance (MDR), was recently obtained from the successful cloning and transfection of the *mdr*1 gene, which codes for a 170 kDa plasmamembrane protein, called P-glycoprotein (Pgp) (Gottesman & Pastan, 1988; Hammond *et al.*, 1989; Juranka *et al.*, 1989; Lincke *et al.*, 1990). The role of this glycoprotein was elucidated by studying the phenotype of cell lines, transfected with *mdr*1 (Gottesman & Pastan, 1989; Hammond *et al.*, 1989; Juranka *et al.*, 1989; Lincke *et al.*, 1990) or selected *in vitro* for resistance to anticancer agents, such as anthracyclines and vinca alkaloids, colchicine and actinomycin D (Biedler *et al.*, 1988; Bradley *et al.*, 1988). Thus Pgp was proven to confer multidrug resistance. Further it was proposed that Pgp functions as a plasma membrane pump for several classes of lipophilic drugs. Evidence for this was based on the predicted amino acid sequence of Pgp, which contained two consensus ATP binding sites, and on the demonstration of ATP-dependent drug binding to Pgp and energy-dependent drug efflux from Pgp/+ cells (Broxterman *et al.*, 1988; Choi *et al.*, 1988; Gros *et al.*, 1986). However, it has been shown that in some Pgp/+ cells with high levels of doxorubicin resistance the net change in cellular accumulation of drug was relatively modest, which was taken as argument against an important role of such a drug export protein in the resistance mechanism in these cells (Siegfried *et al.*, 1983). Recently we showed that in the Pgp/+ cells $2780^{AD}$ and $CH^{R}C5$, the resistance to doxorubicin could be quantitatively accounted for, when, in addition to doxorubicin accumulation, the distribution of doxorubicin between nuclear sites and cytoplasmic localisations was taken into consideration (Schuurhuis *et al.*, 1989). The data suggested that these Pgp/+ cells had developed a resistance mechanism characterised by a decrease in net cellular doxorubicin accumulation, including the nuclear target sites, while accumulation in cytoplasmic structures was relatively unaffected.

We have now investigated whether such a defence mechanism of mammalian cells against certain types of anticancer agents is necessarily dependent on Pgp overexpression and we have therefore especially focussed on low level resistant cells. Hereto we used two series of variants selected by doxorubicin exposure from the human non-small cell lung cancer cell line, SW-1573, which both displayed multidrug resistance.

### Materials and methods

### *Cells and cell culture*

The human non-small cell lung cancer cell line was obtained from Dr H. Joenje (Department of Anthropogenetics, Free University, Amsterdam; Keizer *et al.*, 1989) and was cultured in Dulbecco's modified Eagle's medium + 7.5–10% foetal calf serum (GIBCO Europe, Paisley, Scotland). The resistant sublines were derived by continuous exposure to increasing doxorubicin concentrations: e.g. 2R50 (2R80 etc) means that these cells were continuously cultured in 50 (80 etc) nM doxorubicin, until they were harvested 7–14 days before the experiments by short trypsinisation. There was no significant difference in cell size between all the SW-1573 sublines (diameter SW-1573: $16.2 \pm 1.2\,\mu m$ in nine experiments). Cell cycle distribution as measured with flow cytometry (G1, S and G2 + M phase, resp., expressed as % of total number of cells and determined in two independent experiments) was as follows. SW-1573: 54, 37 and 9, resp.; SW-1573/2R50: 59,33 and 9, resp.; SW-1573/2R120: 62, 20 and 19, resp.; SW-1573/4R50: 41, 40 and 19, resp.; SW-1573/4R120: 54, 28 and 18, resp. Cell doubling times were $22 \pm 1$ (SW-1573), $32 \pm 3$ (SW-1573/2R50), $38 \pm 3$ (SW-1573/2R120), $43 \pm 5$ (SW-1573/2R160), $26 \pm 1$ (SW-1573/4R50) and $28 \pm 2$ (SW-1573/4R120), determined in 3–5 independent experiments (M ± s.d.).

### *Drug cytotoxicity*

Doxorubicin resistance factor (RF) and dose-modifying factor (DMF) were calculated from 50% cell-growth inhibitory

Correspondence: G.J. Schuurhuis.
Received 19 March 1991; and in revised form 12 July 1991.

concentrations of doxorubicin ($IC_{50}$) determined in a cell proliferation assay; $IC_{50}$ of SW-1573 cells was 0.12 ± 0.02 μM (mean ± s.d. of 5 exp., 2 h drug exposure in a waterbath at 37°C in culture medium lacking $NaHCO_3$ but containing 20 mM HEPES). After 2 h exposure to doxorubicin and 32 μM verapamil (for DMF), the cell culture medium was refreshed and after another 4 h with or without verapamil in the same medium the cells were allowed to grow in $NaHCO_3$ containing medium for three cell doubling-times, again in the presence of 32 μM verapamil for DMF determinations (Schuurhuis *et al.*, 1987). The 2 h exposure was carried out in medium lacking $NaHCO_3$ in order to be able to compare the cytotoxicity data directly with drug accumulation and N/C ratio measurements (see below). The $IC_{50}$ value for SW-1573 cells was lower (factor 2) in medium lacking $NaHCO_3$ than in growth medium but no major differences in resistance factors were found when the two media were compared. Resistance factors for the other drugs were determined in a cell proliferation assay by a continuous incubation with drugs as described (Broxterman *et al.*, 1989; Mosman, 1983). A concentration of 32 μM verapamil was used in order to obtain maximal effects on doxorubicin accumulation, cytotoxicity and intracellular drug distribution, since in case low level MDR cells are used these parameters do not differ much from those in sensitive cells. Previously dose-dependent effects of verapamil on these parameters have been shown (Schuurhuis *et al.*, 1987, 1989, 1990).

### *Drug accumulation*

Doxorubicin accumulation and accumulation-enhancement factor by co-incubation with 32 μM verapamil (AEF) were determined by exposure of adhered cells to 0.5 μM [$^{14}$C]-doxorubicin (2 h, 37°C) in culture medium lacking $NaHCO_3$, three rapid cold washes and subsequent trypsinisation of the cells; further procedures were essentially as described (Schuurhuis *et al.*, 1987).

### *Flow cytometry*

For quantification of Pgp, $10^6$ unfixed cells were incubated with the monoclonal antibody MRK-16 (4 μg ml$^{-1}$) or an irrelevant mouse IgG (5 μg ml$^{-1}$) for 1 h in a volume of 200 μl at 20°C. Samples were washed three times with phosphate buffered saline + 1% bovine serum albumin (PBS-BSA) and incubated with 100 μl rabbit-antimouse IgG-fluoresceinisothiocyanaat (100 μg ml$^{-1}$), DAKO immunoglobulins, Copenhagen, Denmark) for 45 min at 20°C. After washing the cells three times with PBS-BSA, the cells were resuspended in 500 μl PBS-BSA and fluorescence was measured with a FACSTAR Plus, Becton Dickinson Medical Systems (Sharon, Ma).

### *Determination of N/C doxorubicin fluorescence ratios*

Trypsinised cells were allowed to adhere on tissue culture petri dishes (Costar, Cambridge, Ma) for 24 h. Cells were incubated with doxorubicin for 1 h under the same conditions as described for drug accumulation and cytotoxicity experiments in Table I and quickly washed with PBS to reduce background fluorescence. PBS was lacking glucose to prevent drug efflux and redistribution. Dox concentrations were chosen to obtain equal net cellular drug amounts in all cell lines (4 μM in SW-1573 cells). Thirty to fifty cells were recorded for each treatment using laserscan microscopy and fluorescence ratios were quantified by delineating nuclei and cytoplasms interactively using digital image analysis as described (Schuurhuis *et al.*, 1989).

### *Determination of the ratio intercalated doxorubicin/non-intercalated fluorescent doxorubicin*

The ratios of intercalated doxorubicin *vs* non-intercalated fluorescent doxorubicin were determined as described (Lankelma *et al.*, 1990). SW-1573 and SW-1573/2R160 cells were loaded for 1 h at 37°C with 4 and 20 μM doxorubicin, respectively, in order to obtain about equal intracellular drug amounts.

### *RNAase protection assay*

Ten μg RNA samples were hybridised with a $^{32}$P-labelled 301 nucleotide human *mdr*1 cDNA specific probe, obtained from F. Baas, Neth. Cancer Inst., and analysed by RNAase protection assay essentially as described (Baas *et al.*, 1990; Zinn *et al.*, 1983). A τ-actin probe was used to control for equal amounts of analysed RNA.

### Results and discussion

Two separate series of resistant cells were selected by continuous exposure to 50, 80, 120 and 160 nM doxorubicin. The cross-resistance pattern of the 2R series has recently been described (Baas *et al.*, 1990; Kuiper *et al.*, 1990); it was shown that all sublines from this series had a decreased doxorubicin and vincristine accumulation, while Pgp expression, which was detectable with a sensitive RNAase protection assay in the parent cell line, was lost during an early selection step, but reappeared, strongly overexpressed, at a later selection step (Baas *et al.*, 1990; Kuiper *et al.*, 1990). In

**Table I** Doxorubicin resistance and accumulation in SW-1573 variants

| *Cell line* | *RF (dox)* | *DMF*[a] *(32 μM Vp)* | *dox accumulation (% of SW-1573)* | *AEF*[b] *(32 μM Vp)* |
|---|---|---|---|---|
| SW-1573 | 1 | 1.6±0.3[d] | 100 | 1.16±0.09[c] |
| 4R50 | 7.7±3.8 | 7.3±1.4[d] | 12.2±0.5[d] | 5.9 ±0.1[c] |
| 4R80 | 10.0±1.2 | 12.8±1.4[d] | 10.2±0.2[d] | 7.4 ±0.7[d] |
| 4R120 | 13.2±1.9 | 10.0±0.9[d] | 10.9±1.0[d] | 5.2 ±0.4[d] |
| 4R160 | 16.7±0 | 16.1±0.9[d] | 7.1±0.2[d] | 10.0 ±1.1[c] |
| 2R50[e] | 6.9±0.3 | 2.5±0.1[c] | 34.0±6.0[d] | 1.3 ±0.1 |
| 2R80[e] | 9.4±2.0 | 5.5±1.4[d] | 35.4±2.5[d] | 1.3 ±0.2 |
| 2R120[e] | 11.1±2.8 | 6.3±0.4[d] | 41.9±8.0[d] | 1.4 ±0.2 |
| 2R160 | 63 ±12 | 17.2±1.3[d] | 4.7±0[d] | 9.3 ±1.7[d] |

[a]DMF, dose modifying factor = $IC_{50}$ minus Vp/$IC_{50}$ plus Vp. [b]AEF, accumulation enhancement factor = drug accumulation plus Vp/drug accumulation minus Vp. Data are means ± s.d. of 2–3 experiments each performed at least in duplicate (cytotoxicity) or triplicate (accumulation). [c,d] Significantly different from 1 (DMF), from SW-1573 (-Vp) levels (fourth column) or from accumulation -Vp (fifth column): [c]$P<0.02$; [d]$P<0.01$ (Student's *t*-test). [e]non-Pgp cell lines, as measured with RNAase protection assay (Baas *et al.*, 1990; Zinn *et al.*, 1983). Vp, verapamil.

**Table II** Cross-resistance in SW-1573 variants

| *Cell line* | *RF (dauno)* | *RF (vincr)* | *RF (gramD)* | *RF (etoposide)* | *Pgp*[a] |
|---|---|---|---|---|---|
| SW-1573 | 1 | 1 | 1 | 1 | 1.08±0.05 |
| 4R50 | 3.0±1.3 | 213± 80 | 161±53 | 6.6±2.0 | 21.4 ±4.2 |
| 4R80 | 3.4±1.6 | 222± 76 | 185±70 | 5.0±2.1 | 37.6 ±4.3 |
| 4R120 | 4.9±3.5 | 299± 55 | 149±45 | 5.2±1.2 | 20.8 ±2.4 |
| 4R160 | 6.6±3.7 | 413±108 | 202±55 | 5.4±2.3 | 22.4 ±5.4 |
| 2R50[b] | 3.3±0.4 | 5.8±1.1 | 3.2±1.1 | 14± 6 | 1.10±0.15 |
| 2R80[b] | 5.9±1.6 | 16± 3 | 3.1 ($n$ = 1) | 21±17 | ND |
| 2R120[b] | 3.7±0.1 | 17± 3 | 2.3±1.7 | 45±13 | 1.02±0 |
| 2R160 | 35±9 | 480±170 | 146±33 | 120±36 | 20.4 ±8.2 |

Resistance factors (RF) were determined in a continuous incubation assay with drug as described (Broxterman *et al.*, 1989; Mosmann, 1983). Data are means ± s.d. from 3–4 separate experiments. The RF data for daunorubicin, vincristine, gramicidin D and etoposide for the 2R series are from Kuiper *et al.*, 1990 and are shown for comparison. [a]Data are given as mean fluorescence of MRK-16, divided by mean fluorescence IgG (mean ± s.d. of 2 experiments). Fluorescence ratio for $2780^{AD}$ cells (Schuurhuis *et al.*, 1987) was 37 (mean of seven experiments). [b]Pgp negative cell lines (see Table I). ND, not determined.

**Figure 1** *mdr*1 overexpression is an all or non phenomenon in the present cell lines. The RNAase protection assay was carried out as described under Materials and methods. From left to right bands corresponding to RNA from SW-1573, 4R50, 4R80, 4R120 and 4R160 are shown. The parent SW-1573 cells have a just detectable signal, which varies somewhat with proliferation state of the cells (Baas *et al.*, 1990). It was shown before that the similarity in intensity was not due to saturation phenomena because that a further increase of intensity occurs with a further increase of drug selection pressure (Baas *et al.*, 1990).

a separate selection with doxorubicin, the 4R series was derived (Tables I and II) which appeared to have overexpression of Pgp from the first selection step on, as determined at protein level with the monoclonal antibody MRK-16 (Table II) and with a RNAase protection assay using a *mdr*1 specific probe (Figure 1). Remarkably the 4R50, 4R120, 4R160 as well as the 2R160 cells have similar amounts of Pgp, while the 4R80 cells for unknown reasons have higher amounts (Table II). In separate experiments it was shown that cells with a higher degree of resistance ($2780^{AD}$, Schuurhuis *et al.*, 1987) have a higher amount of Pgp (see legends of Table II), indicating non-saturability of the method used. Both the 2R and 4R series display a MDR-like phenotype with, relative to doxorubicin and daunorubicin resistance (Tables I and II), high vincristine and gramicidin D resistance in Pgp/+ cells and high etoposide resistance in Pgp/− cells (Table II). Doxorubicin resistance could be modulated by coincubation with verapamil in 4R.Pgp/+ cells to a large extent (Table I). In 2R.Pgp/− cells, however, the resistance modulation with verapamil was less effective (Table I). Both the 2R and 4R series show an impairment of doxorubicin accumulation, which was more prominent, however, in the 4R series (Table I). Especially in the 2R series the accumulation defect would not be sufficient to fully account for the observed resistance factors. Doxorubicin accumulation, like doxorubicin resistance, could be modulated more effectively in the Pgp/+ cells (Table I). Thus, while interaction of verapamil with Pgp seems to allow an effective modulation of doxorubicin resistance and accumulation, it does not prove that doxorubicin resistance and impairment of accumulation in these Pgp/+ cells is directly caused by Pgp.

We have shown before by a quantitative approach using laserscan microscopy and image analysis that a decrease in doxorubicin fluorescence nucleus/cytoplasm (N/C) ratio could be measured in the low level *mdr* Pgp/+ cell line 8226/dox 4 (Broxterman *et al.*, 1990). Those data suggested a correlation of doxorubicin fluorescence N/C ratio with Pgp expression. The present SW-1573 experimental system allowed us to study the doxorubicin fluorescence N/C ratio's in cells with increasing levels of multidrug resistance in Pgp/+ as well as Pgp/− cells, derived from the same parent cells.

Figure 2 shows a decrease in N/C ratio with increasing selection pressure in Pgp/+ as well as in Pgp/− cells. Interestingly, N/C ratios were slightly lower in Pgp/− cells compared to Pgp/+ cells at the same levels of resistance, while on the other hand doxorubicin accumulation was less in the Pgp/+ cells (Table I). This suggests that the relative contribution of decreased drug accumulation and altered drug

**Figure 2** Doxorubicin fluorescence nucleus/cytoplasm (N/C) ratios as a function of doxorubicin resistance in Pgp/+ and Pgp/− cells. Fluorescence N/C ratios were determined as described under Materials and methods. Data are from three independent experiments each performed in duplicate (means ± s.d.).

**Table III** Effect of verapamil on doxorubicin fluorescence ratios in SW-1573 variants

| *Cell line* | *N/C* | *N/C ( + verapamil)* |
|---|---|---|
| SW-1573 | 3.77 ± 0.18 | 3.87 ± 0.44 [1.0] |
| 4R50 | 2.60 ± 0.18[b] | 3.27 ± 0.25[c] [1.3] |
| 4R80 | 1.89 ± 0.37[b] | 4.02 ± 0.18[e] [2.1] |
| 4R120 | 2.02 ± 0.17[b] | 3.04 ± 0.10[d] [1.5] |
| 4R160 | 1.16 ± 0.24[b] | 2.61 ± 0.11[e] [2.3] |
| 2R50[a] | 2.18 ± 0.16[b] | 1.70 ± 0.03 [0.8] |
| 2R80[a] | 1.72 ± 0.24[b] | 1.81 ± 0.23 [1.1] |
| 2R120[a] | 1.75 ± 0.13[b] | 1.77 ± 0.37 [1.0] |
| 2R160 | 0.67 ± 0.12[b] | 2.39 ± 0.64[e] [3.6] |

N/C ratios (doxorubicin fluorescence in nucleus/doxorubicin fluorescence in cytoplasm) were measured as described for Figure 2. [a] Pgp negative cell lines. [b]Significantly different from 3.77 ($P < 0.01$, Student's *t*-test). [c,d,e]Significantly different from N/C ratios minus verapamil: [c]$P < 0.05$; [d]$P < 0.02$; [e]$P < 0.01$. Values in brackets represent factor increase of N/C ratios by verapamil. Data are from 2–6 independent experiments each performed in duplicate.

distribution to the resistance phenotype may differ depending on the presence of Pgp. The fact that 4R160 cells have a lower N/C ratio than e.g. 4R50 cells, despite similar amounts of Pgp, indicates that the non-Pgp resistance mechanism might also be present in the 4R cells. However, it cannot be excluded that the high resistance to VP-16 in Pgp/− cells would be caused in part by additional changes such as an altered topoisomerase II activity (Baas et al., 1990).

Again, like for doxorubicin accumulation, for an effective modulation of doxorubicin fluorescence N/C ratio by verapamil the presence of Pgp seems to be a prerequisite (Table III, compare e.g. 2R120 with 4R120 cells). Remarkably, verapamil-induced changes in accumulation (Table I) together with changes in N/C ratios (Table III) can account for resistance modulation (DMF, Table I) in Pgp/+ cells but not in Pgp/− cells, which leaves the possibility of additional actions of verapamil which affect doxorubicin cytotoxicity. In the 4R series the 4R80 cells show a somewhat exceptional behaviour: the amount of Pgp as estimated with flow cytometry is higher than in the other 4R cells (Table II). In line with this stimulation of doxorubicin accumulation as well as reversal of resistance by verapamil was more prominent in the 4R80 cells (Table I). Also the N/C ratio in 4R80 cells was relatively low, while its modification with verapamil was relatively high (Table III).

In this study doxorubicin fluorescence N/C ratios are used operationally to probe mechanisms of drug resistance, related to changes in intracellular drug distribution. These ratios as such do not reflect the actual concentrations of doxorubicin in each compartment, since they do not take into account the quenching of doxorubicin fluorescence at several different localisations of the drug: quenching of doxorubicin fluorescence due to DNA intercalation has been estimated at 95% (Chaires et al., 1982), while cytoplasmic fluorescence may be largely unaffected (Budge & Tritton, 1985; Tarasiuk et al., 1989). We have used an independent technique to show that N/C ratio changes reflect changes in the actual compartmental amounts of doxorubicin. With this technique the ratio intercalated drug/non-intercalated fluorescent drug can be measured (Lankelma et al., 1990). It was found that this ratio was $4.2 \pm 0.6$ in SW-1573 cells and $1.2 \pm 0.2$ in SW-1573/2R160 cells (M ± s.e.m. of two independent experiments). N/C ratio changes might also be found if certain drug binding sites in the cell are saturated at the relatively high drug concentrations which were used in the fluorescence assays. This is unlikely, however, since N/C ratios were shown to be independent of doxorubicin concentrations in the medium (J.H.M. de Lange, N.W. Schipper, G.J. Schuurhuis et al., manuscript submitted).

The molecular mechanism(s) responsible for the induction of decreases in doxorubicin N/C ratio are not yet known; in the SW-1573 cell lines the presence of Pgp is not required for this decrease to occur. One explanation would be the presence of a pump protein present at vesicular membranes oriented to pump drug inside such vesicles. However, in the case of Pgp, the antigenic determinant of MRK-16 could be detected only on the Golgi stack but not in other vesicles (Willingham et al., 1987). Moreover, similar amounts of Pgp, as estimated with MRK-16 binding, do not seem to correlate with resistance factor or N/C ratios (compare e.g. 2R160 and 4R160 cells in the Tables I–III). Thus, even after the induction of Pgp overexpression, the early, non-Pgp mediated mechanism, may still be operative. Since cytoplasmic pH was elevated in some resistant SW-1573 isolates, compared to SW-1573 parent cells as measured in medium without bicarbonate (Keizer & Joenje, 1989), one possibility to be considered is that anthracyclines are forced to accumulate to a higher extent in an acidic vesicular compartment in the cell (Beck, 1987; Keizer et al., 1989; Sehested et al., 1987). Since even in steady-state the 2R.Pgp/− cells accumulated less doxorubicin (Table I) or vincristine (Kuiper et al., 1990), an active extrusion process could be present. Active drug extrusion processes for different types of drugs, not related to Pgp overexpression, have in fact been reported recently (Henderson & Tsuji, 1990; Hindenburg et al., 1989; McGrath et al., 1989a,b). In such highly resistant cells anthracycline resistance could also be related to differences in drug binding to nuclear and cytoplasmic bindings sites (Hindenburg et al., 1989). Since different independently selected, lowly resistant isolates from SW-1573 cells showed the Pgp/− multidrug resistance (Baas et al., 1990), which now has been shown to be related to decreased doxorubicin N/C ratio's, a mutation in one gene product could be responsible for the observed resistance pattern in Pgp/− cells. The remarkably low resistance factors of 4R160 compared to 2R160 for both anthracyclines and etoposide, despite a similar Pgp expression further suggests that such a mechanism still prevails in the resistance of 2R160 Pgp/+ cells for these drugs. The combined measurement of doxorubicin accumulation and fluorescence N/C ratio's in tumour cells, isolated directly from patient's tumours would allow to investigate the role of drug transport resistance in failure of chemotherapy. Further, applying immunohistochemical staining techniques after N/C ratio measurements would allow a direct comparison of functional changes (reflected by N/C ratio changes) and the presence of resistance-related antigenic determinants (e.g Pgp) in individual cells.

This study was supported by the Dutch Cancer Society (grant IKA-VU 88-22) and by a grant from the Bristol-Myers Squibb Company. H.J.B. is a fellow of the Royal Netherlands Academy of Arts and Sciences. We thank H.S Mülder for determination of the ratios intercalated drug/non-intercalated drug. Dr P. Borst and Dr F. Baas are acknowledged for their stimulating discussions of this work. We thank Dr T. Tsuruo for the generous supply of MRK-16.

### References

BAAS, F., JONGSMA, A.P.M., BROXTERMAN, H.J. & 7 others (1990). Non-P-glycoprotein mediated mechanism for multidrug resistance precedes P-glycoprotein expression during *in vitro* selection for doxorubicin resistance in a human lung cancer cell line. *Cancer Res.*, **50**, 5392.

BECK, W.T. (1987). The cell biology of multiple drug resistance. *Biochem. Pharmacol.*, **36**, 2879.

BIEDLER, J.L., CHANG, T-d., SCOTTO, K.W., MELERA, P.W. & SPENGLER, B.A. (1988). Chromosomal organization of amplified genes in multidrug-resistant Chinese hamster cells. *Cancer Res.*, **48**, 3179.

BRADLEY, G., JURANKA, P.F. & LING, V. (1988). Mechanism of multidrug resistance. *Biochim. Biophys. Acta*, **948**, 87.

BROXTERMAN, H.J., PINEDO, H.M., KUIPER, C.M., KAPTEIN, L.C.M., SCHUURHUIS, G.J. & LANKELMA, J. (1988). Induction of verapamil of a rapid increase in ATP consumption in multidrug-resistant tumor cells. *FASEB J.*, **2**, 2278.

BROXTERMAN, H.J., PINEDO, H.M., KUIPER, C.M. & 7 others (1989). Immunohistochemical detection of P-glycoprotein in human tumour cells with a low degree of drug resistance. *Int. J. Cancer*, **43**, 340.

BROXTERMAN, H.J., SCHUURHUIS, G.J., LANKELMA, J., BAAK, J.P.A. & PINEDO, H.M. (1990). Towards functional screening for multidrug resistant cells in human malignancies. In *Drug Resistance: Mechanisms and Reversal*, Mihich, E. (ed.), p. 309, John Libbey CIC: Roma.

BUDGE, T.G. & TRITTON, T.R. (1985). Location and dynamics of anthracyclines bound to unilamellar phosphatidylcholine vesicles. *Biochemistry*, **24**, 5972.

CARL, J. (1989). Drug-resistance patterns assessed from tumor marker analysis. *J. Natl Cancer Inst.*, **81**, 1631.

CHAIRES, J.B., DATTAGUPTA, N. & CROTHERS, D.M. (1982). Studies on interaction of anthracycline antibiotics and deoxyribonucleic acid: equilibrium binding studies on interaction of daunomycin with deoxyribonucleic acid. *Biochemistry*, **21**, 3933.

CHOI, K., CHEN, C-j., KRIEGLER, M. & RONINSON, I.B. (1988). An altered pattern of cross-resistance in multidrug-resistant human cells results from spontaneous mutations in the mdr1 (P-glycoprotein) gene. *Cell*, **53**, 519.

COLDMAN, A.J. & GOLDIE, J.H. (1985). Role of mathematical modeling in protocol formulation in cancer chemotherapy. *Cancer Treat. Rep.*, **69**, 1041.

GOTTESMAN, M.M. & PASTAN, I. (1988). Resistance to multiple chemotherapeutic agents in human cancer cells. *Tr. Pharmacol. Sci.*, **9**, 54.

GROS, P., CROOP, J. & HOUSMAN, D. (1986). Mammalian multidrug resistance gene: complete cDNA sequence indicates strong homology to bacterial transport proteins. *Cell*, **47**, 371.

HAMMON, J.R., JOHNSTONE, R.M. & GROS, P. (1989). Enhanced efflux of [$^3$H] vinblastine from Chinese hamster ovary cells transfected with a full-length complementary DNA clone for the mdr1 gene. *Cancer Res.*, **49**, 3867.

HENDERSON, G.B. & TSUJI, J.M. (1990). Identification of cholate as a shared substrate for the unidirectional efflux systems for metrotrexate in L1210 mouse cells. *Biochem. Biophys. Acta*, **1051**, 60.

HINDENBURG, A.A., GERVASONI, J.E., KRISHNA, S. & 6 others (1989). Intracellular distribution and pharmacokinetics of daunorubicin in anthracycline-sensitive and -resistant HL-60 cells. *Cancer Res.*, **49**, 4607.

JURANKA, P.F., ZASTAWNY, R.L. & LING, V. (1989). P-glycoprotein: multidrug-resistance and a superfamily of membrane-associated transport proteins. *FASEB J.*, **3**, 2583.

KEIZER, H.G. & JOENJE, H. (1989). Increased cytosolic pH in multidrug-resistant human lung tumor cells: effect of verapamil. *J. Natl Cancer Inst.*, **81**, 706.

KEIZER, H.G., SCHUURHUIS, G.J., BROXTERMAN, H.J. & 5 others (1989). Correlation of multidrug resistance with decreased drug accumulation, altered subcellular drug distribution, and increased P-glycoprotein expression in cultured SW-1573 human lung tumor cells. *Cancer Res.*, **49**, 2988.

KUIPER, C.M., BROXTERMAN, H.J., BAAS, F. & 5 others (1990). Drug transport variants without P-glycoprotein overexpression from a human squamous lung cancer cell line after selection with doxorubicin. *J. Cell Pharmacol.*, **1**, 35.

LANKELMA, J., MÜLDER, H.S., VAN MOURIK, F., WONG FONG SANG, H.W., KRAAYENHOF, R. & VAN GRONDELLE, R. (1991). Cellular daunomycin fluorescence in multidrug resistant 2780$^{AD}$ cells and its relation to cellular drug localisation. *Biochim. Biophys. Acta*, **1093**, 147.

LINCKE, C.R., VAN DER BLIEK, A.M., SCHUURHUIS, G.J., VAN DER VELDE-KOERTS, T., SMIT, J.J.M. & BORST, P. (1990). Multidrug resistance phenotype of human BRO melanoma cells transfected with a wild-type human mdr1 complementary DNA. *Cancer Res.*, **50**, 1779.

MCGRATH, T., LATOUD, C., ARNOLD, S.T., SAFA, A.R., FELSTED, R.L. & CENTER, M.S. (1989*a*). Mechanisms of multidrug resistance in HL60 cells. Analysis of resistance associated membrane proteins and levels of *mdr* gene expression. *Biochem. Pharmacol.*, **38**, 3611.

MCGRATH, T., MARQUARDT, D. & CENTRE, M.S. (1989*b*). Multiple mechanisms of adriamycin resistance in the human leukemia cell line CCRF-CEM. *Biochem. Pharmacol.*, **38**, 497.

MOSMANN, T. (1983). Rapid colometric assay for cellular growth and survival: application to proliferation and cytotoxicity assays. *J. Immunol. Meth.*, **65**, 55.

SCHUURHUIS, G.J., BROXTERMAN, H.J., CERVANTES, A. & 5 others (1989). Quantitative determination of factors contributing to doxorubicin resistance in multidrug-resistant cells. *J. Natl Cancer Inst.*, **81**, 1887.

SCHUURHUIS, G.J., BROXTERMAN, H.J., PINEDO, H.M. & 5 others (1990). The polyoxyethylene castor oil Cremophor EL modifies multidrug resistance. *Br. J. Cancer*, **62**, 591.

SCHUURHUIS, G.J., BROXTERMAN, H.J., VAN DER HOEVEN, J.J.M., PINEDO, H.M. & LANKELMA, J. (1987). Potentiation of doxorubicin cytotoxicity by the calcium antagonist bepridil in anthracycline-resistant and -sensitive cell lines. A comparison with verapamil. *Cancer Chemother. Pharmacol.*, **20**, 285.

SEHESTED, M., SKOVSGAARD, T., VAN DEURS, B. & WINTER-NIELSEN, H. (1987). Increased plasma membrane traffic in daunorubicin resistant P388 leukaemic cells. *Br. J. Cancer*, **56**, 747.

SIEGFRIED, J.M., TRITTON, J.R. & SARTORELLI, A.C. (1983). Comparison of anthracycline concentrations in S180 cell lines of varying sensitivity. *Eur. J. Cancer Clin. Oncol.*, **19**, 1133.

SKIPPER, H.E. (1986). On mathematical modeling of critical variables in cancer treatment. Goals: better understanding of the past and better planning in the future. *Bull. Math. Biol.*, **48**, 253.

TARASIUK, J., FREZARD, F., GARNIER-SUILLEROT, A. & GATTEGNO, L. (1989). Anthracycline incorporation in human lymphocytes. Kinetics of uptake and nuclear concentration. *Biochim. Biophys. Acta*, **1013**, 109.

WILLINGHAM, M.C., RICHERT, N.D., CORNWELL, M.M. & 4 others (1987). Immunocytochemical localization of P170 at the plasma membrane of multidrug-resistant human cells. *J. Histochem. Cytochem.*, **35**, 1451.

ZINN, K., DIMAIO, D. & MANIATIS, T. (1983). Identification of two distinct regulatory regions adjacent to the human b-interferon gene. *Cell*, **34**, 865.